# Examination of annulus fibrosus and nucleus pulposus in cervical and lumbar intervertebral disc herniation patients by scanning acoustic microscopy, scanning electron microscopy and energy dispersive spectroscopy

**DOI:** 10.1039/d3ra07195b

**Published:** 2024-01-15

**Authors:** Bukem Tanoren, Beste Dipcin, Selcuk Birdogan, Mehmet Burcin Unlu, Cagatay Ozdol, Kamrhan Aghayev

**Affiliations:** a Acibadem University, Faculty of Engineering and Natural Sciences, Department of Natural Sciences Istanbul Turkey bukem.tanoren@acibadem.edu.tr +90 216 500 4156 +90 216 576 5076; b Acibadem University, Faculty of Engineering and Natural Sciences, Department of Molecular Biology and Genetics Istanbul Turkey; c Sabanci University SUNUM Nanotechnology Research and Application Center Istanbul Turkey; d Bogazici University, Department of Physics Istanbul Turkey; e Antalya Education and Research Hospital Istanbul Turkey; f Esencan Hospital Istanbul Turkey

## Abstract

Intervertebral disc herniation (IVDH) is observed in humans as a result of the alteration of annulus fibrous (AF) and nucleus pulposus (NP) tissue compositions in intervertebral discs. In this study, we studied the feasibility of scanning acoustic microscopy (SAM), scanning electron microscopy (SEM) and energy dispersive spectroscopy (EDS) in characterizing the herniated segments of AF and NP tissues from male and female patients. SAM determined the acoustic property variations in AF and NP tissues by calculating the acoustic impedance values of samples of 15 patients. SEM obtained higher resolution images and EDS made elemental analysis of the specimen. Consequently, we suggest that these techniques have the potential to be combined for the investigation and removal of the disrupted AF and NP tissues with micrometer resolution in clinics.

## Abstract 2

Scanning acoustic microscopy, scanning electron microscopy and energy dispersive spectroscopy of annulus fibrous and nucleus pulposus tissues from patients with intervertebral disc herniation were performed, to analyse and determine the acoustic property variations in the tissues.

## Graphical abstract

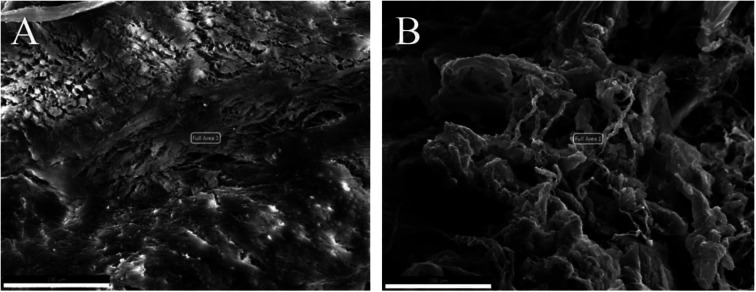

## 1 Introduction

Intervertebral disc herniation (IVDH) is one of the major causes of back pain and disability with high morbidity.^1,2^ The human spine consists of five specific sections – cervical, thoracic, lumbar, sacrum, and coccyx.^3^ Cervical, thoracic and lumbar sections are mobile due to the presence of intervertebral discs which are joints in between adjacent vertebrae. An intervertebral disc consists of nucleus pulpous and annulus fibrosus. Nucleus pulposus (NP) is a gelatinous core of the disc and is surrounded by thick fibrous annulus fibrosus (AF).^3^ IVDH is caused by degeneration of NP leading to loss of integrity, fragmentation and subsequent herniation of NP material through disrupted AF into the spinal canal. Although the exact mechanism of NP degeneration is mostly unknown it is known that several factors such as repetitive trauma with high mechanical load (wear and tear) and aging contribute to pathophysiology of intervertebral disc degeneration.^4,5^ The loss of integrity may be influenced or accelerated by the disorganization in some extracellular matrix (ECM) proteins such as the collagen fibers.^6,7^ Also, it is known that herniated intervertebral discs might become calcified, especially in the thoracic spine and rarely in other segments making the treatment of the thoracic herniation more difficult.^8^ The symptoms of disc herniation depend on the location and are mainly attributed to the compression of the spinal cord and/or nerve roots. Typically they include pain in the corresponding level of the spine (neck pain, back pain or lower back pain) radiating to upper or lower extremities. Numbness and weakness in arms and legs can also develop if neural structures are compressed to a significant degree.

The diagnosis of the IVDH is mainly based on medical imaging instruments that are considered to be relatively expensive.^9^ Magnetic resonance imaging (MRI) is one of them and the most effective one due to the fact that it has good soft-tissue visualization capacity and besides, patients are not exposed to radiation.^9,10^ Computed tomography (CT) (contrast-enhanced CT, non-contrast CT, or multi-detector CT) is used for detecting intervertebral disc herniation, since it can give information about the size and the shape of the herniated disc.^9,11^ Myelography is another method that is old but useful for the diagnosis of root compression.^12^ Also, X-ray imaging is used for the cervical or lumbar disc herniation, since it is a cheap and easily accessible method.^9^ Using CT, myelography and X-ray imaging modalities for the diagnosis of cervical or lumbar disc herniation, patients are subjected to radiation.^9,11–13^

Scanning acoustic microscopy (SAM) provides information, with micrometer resolution, about the morphology and mechanical properties of biological tissues simultaneously. Focused high-frequency ultrasound is used in SAM with major advantages of high speed in obtaining the two-dimensional images and immediate scanning of the specimen without special preparation and staining. As a result, either the speed of sound (SOS) in specimen tissues^14–18^ or acoustic impedance^19,20^ of samples can be calculated and mapped in 2-dimensions.

Scanning electron microscopy (SEM) is another imaging tool that can characterize samples like tissue sections, cells or nanoparticles by attaining information about the morphology, structure and composition.^21^ Images are obtained by the detection of a variety of signals such as secondary electrons and backscattered electrons that are frequently used for biological samples' imaging, alongside X-rays and cathodoluminescence.^22^ Energy-dispersive spectroscopy (EDS) is used for the semi-quantitative and qualitative analysis of the chemical elements of samples by the analysis of two types of radiations which are continuous radiation that results in the formation of the background of the measurement and the characteristic radiation of a specific wavelength resulting in the detection of the elemental composition.^23–25^

We aimed to evaluate intervertebral disc herniation by examining AF and NP tissues of herniated human cervical and lumbar discs. We characterized the AF and NP tissues by using scanning acoustic microscopy (SAM), scanning electron microscopy (SEM) and energy-dispersive spectroscopy (EDS). SAM provided information on the structural and mechanical properties of samples with micrometer resolution, while, SEM provided morphological information about the herniated tissues with higher resolution. EDS provided chemical information about the herniated AF and NP tissues by element composition analysis. Consequently, we assume that combining these techniques in clinics will help surgeons to designate the altered AF and NP tissues of herniation patients with micrometer resolution, which will minimize the possibility of new herniation formation due to a segment not excised during the surgery.

## 2 Results

### 2.1 Scanning acoustic microscopy results

AF and NP tissues of 15 patients were sliced cross-sectionally for SAM studies. Table 1 shows the average acoustic impedance values of all the tissues examined. 9 patients were female and 6 patients were male of varying ages. Each acoustic impedance value in Table 1 is the average measured over the complete specimen.

**Table 1:** Average impedance values of AF and NP tissues obtained from 15 patients' intervertebral discs from different parts of the spinal cord. 9 female and 6 male patients of varying ages were studied for the correlation of gender and position of the disc on AF and NP tissues. Disc positions are as follows: C3-4 (between the third and fourth cerebral vertebrae), C6-7 (between the sixth and seventh cerebral vertebrae), L3-4 (between the third and fourth lumbar vertebrae), L4-5 (between the third and fourth lumbar vertebrae), L5-S1 (between the fifth lumbar and first sacral vertebrae)

Patients (gender, disc position)	Acoustic impedance of AF	Acoustic impedance of NP
1, male, C6-7	1.578 ± 0.025	1.590 ± 0.030
2, female, C6-7	1.625 ± 0.039	1.533 ± 0.066
3, male, L5-S1	1.597 ± 0.024	1.589 ± 0.044
4, female, L5-S1	1.617 ± 0.037	1.551 ± 0.026
5, male, L3-4	1.587 ± 0.021	1.601 ± 0.015
5, male, L4-5	1.659 ± 0.096	1.578 ± 0.046
6, female, L3-4	1.558 ± 0.031	1.599 ± 0.021
7, female, L4-5	1.557 ± 0.036	1.564 ± 0.014
8, male, C3-4	1.639 ± 0.046	1.594 ± 0.017
9, female, L4-5	1.577 ± 0.017	1.566 ± 0.030
10, female, L5-S1	1.574 ± 0.040	1.608 ± 0.030
11, female, L5-S1	1.595 ± 0.038	1.571 ± 0.059
12, female, L5-S1	1.673 ± 0.018	1.743 ± 0.029
13, female, L3-4	1.620 ± 0.041	1.623 ± 0.022
13, female, L4-5	1.583 ± 0.020	1.544 ± 0.035
13, female, L5-S1	1.632 ± 0.057	1.591 ± 0.028
14, male, L4-5	1.591 ± 0.027	1.569 ± 0.030
15, male, L3-4	1.710 ± 0.040	1.586 ± 0.023

**Fig. 1:**
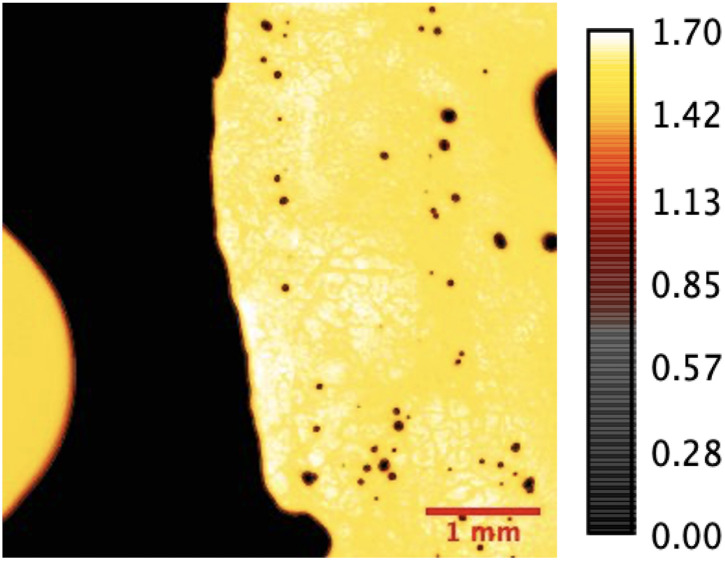
Acoustic impedance map of a male NP tissue sample (patient 5). Unit of the bar is MRayl.

The acoustic impedance maps of the herniated AF and NP tissues are obtained with SAM in acoustic impedance (AI) mode. SAM images were constructed by collecting the reflections of ultrasound signals both from the reference and front surfaces of the slices. Fig. 1 shows the acoustic impedance distribution of the nucleus pulposus tissue sample obtained from a male patient and is an example of the images obtained in AI mode. MATLAB program was used to map acoustic impedance values for each sample.

**Fig. 2:**
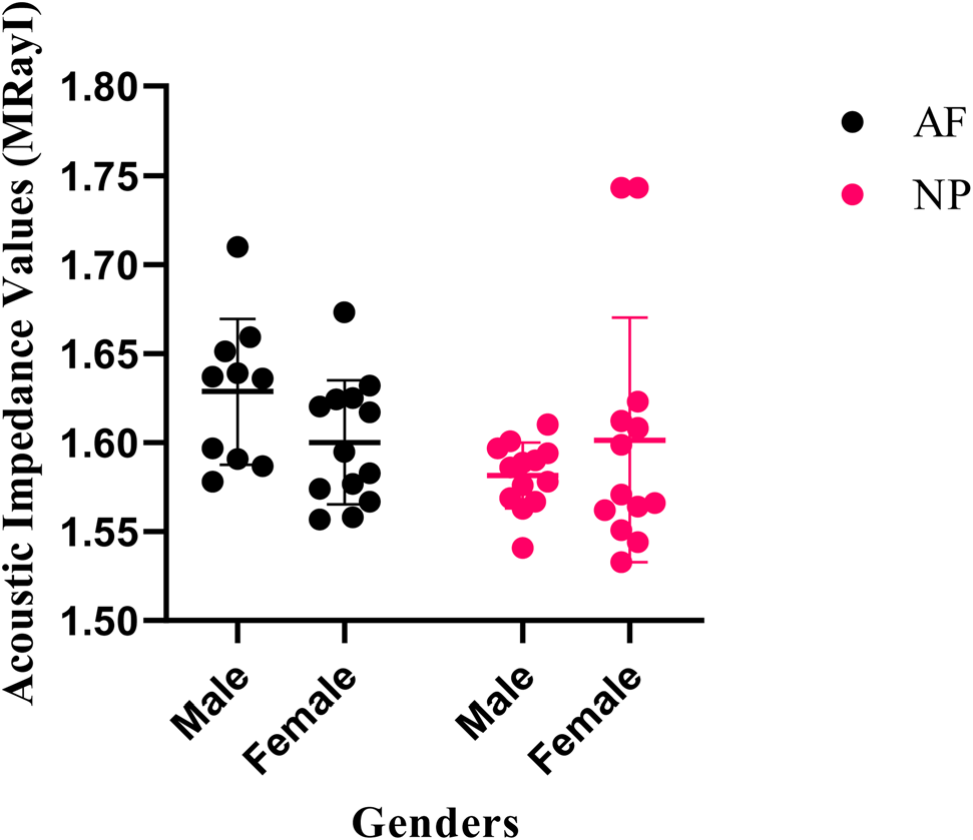
Acoustic impedance values of male and female AF and NP tissues with their uncertainty values.

Fig. 2 shows the acoustic impedance values of male and female annulus fibrosus (AF) and nucleus pulposus (NP) tissues individually with their uncertainty values. The acoustic impedance values are presented in a scatter graph generated by the GraphPad (Prism8) software.

### 2.2 Scanning electron microscopy and energy dispersive spectroscopy results

AF and NP tissue samples are placed inside the microscope for both SEM and EDS.

**Fig. 3:**
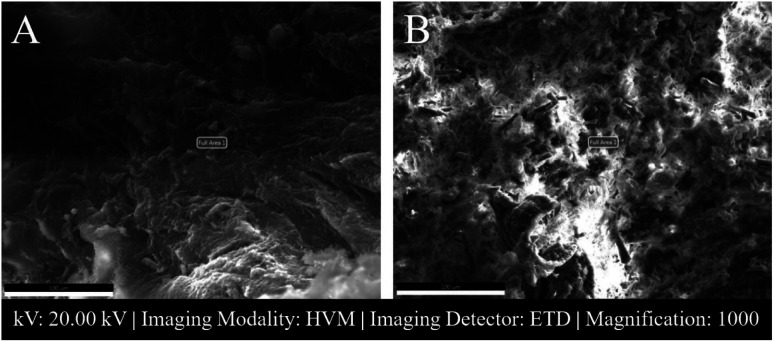
SEM images of male NP tissues. Scale: 100 μm. (A) Male C6-7 NP tissue (patient 1) with low atomic% calcium. (B) Male L3-4 (between the third and fourth lumbar vertebrae) NP tissue (patient 5) that had high atomic% calcium. Full Area 1 shows the area where EDS analysis was done. HVM: High Vacuum Mood, ETD: Everhart–Thorley detector.

Fig. 3 shows SEM images of 2 male NP tissues with low and high atomic% calcium.

**Fig. 4:**
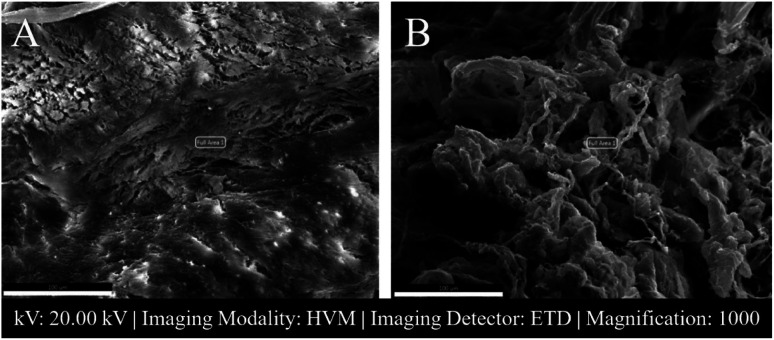
SEM images of female C6-7 intervertebral disc tissues (patient 2). Scale: 100 μm. (A) AF tissue. (B) NP tissue. Full area 1 shows the area where EDS analysis was done. HVM: High Vacuum Mood, ETD: Everhart–Thorley detector.

In Fig. 4 SEM images of a female patient's AF and NP tissues of C6-7 (between the sixth and seventh cerebral vertebrae) intervertebral disc are shown.

**Fig. 5:**
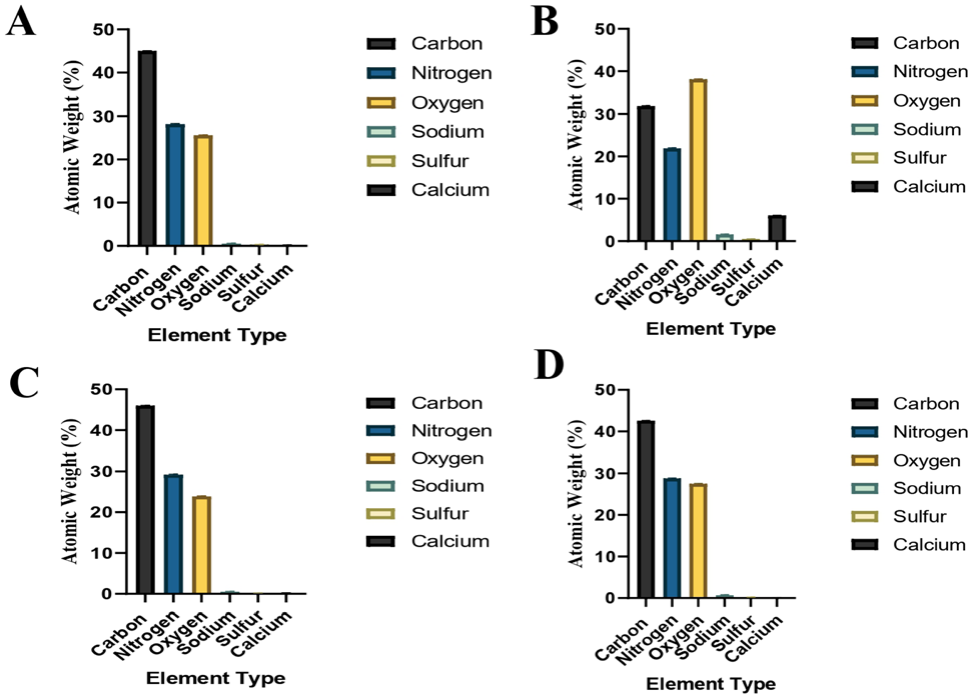
Elemental compositions of the samples that were also investigated with SEM. (A) Male C6-7 NP tissue's elemental composition (Fig. 3A shows its SEM image), (B) male L3-4 NP tissue's elemental composition (Fig. 3B shows its SEM image), (C) female C6-7 AF tissue's elemental composition (Fig. 4A shows its SEM image), (D) female C6-7 NP tissue's elemental composition (Fig. 4B shows its SEM image).

The elemental compositions of the tissues, that were also investigated with SEM as in Fig. 3 and 4, are presented in Fig. 5.

Table 2 shows atomic percentage results of elements of AF and NP tissues with their uncertainty values obtained by energy dispersive spectroscopy (EDS) analysis.

**Table 2:** Atomic percentage (%) results of elements of annulus fibrosus (AF) and nucleus pulposus (NP) tissues with their uncertainty values obtained by energy dispersive spectroscopy (EDS) analysis

Patients (gender, tissue)	Atomic% carbon	Atomic% nitrogen	Atomic% oxygen	Atomic% sodium	Atomic% sulfur	Atomic% calcium
1, male, AF, C6-7	45.0 ± 0.054	29.1 ± 0.103	24.9 ± 0.103	0.6 ± 0.098	0.2 ± 0.026	0.2 ± 0.036
1, male, NP, C6-7	45.0 ± 0.055	28.2 ± 0.102	25.6 ± 0.102	0.5 ± 0.104	0.3 ± 0.023	0.2 ± 0.031
2, female, AF, C6-7	46.0 ± 0.053	29.2 ± 0.106	23.9 ± 0.105	0.5 ± 0.105	0.2 ± 0.031	0.2 ± 0.054
2, female, NP, C6-7	42.6 ± 0.055	28.8 ± 0.100	27.5 ± 0.102	0.7 ± 0.100	0.2 ± 0.027	0.1 ± 0.033
3, male, AF, L5-S1	45.0 ± 0.052	29.7 ± 0.104	24.7 ± 0.104	0.3 ± 0.115	0.1 ± 0.030	0.2 ± 0.039
3, male, NP, L5-S1	42.9 ± 0.057	28.8 ± 0.101	26.6 ± 0.102	0.6 ± 0.097	0.4 ± 0.023	0.7 ± 0.023
4, female, AF, L5-S1	45.5 ± 0.051	30.7 ± 0.102	22.8 ± 0.103	0.6 ± 0.088	0.2 ± 0.030	0.3 ± 0.041
4, female, NP, L5-S1	45.4 ± 0.055	29.6 ± 0.100	23.6 ± 0.101	0.7 ± 0.083	0.4 ± 0.021	0.2 ± 0.037
5, male, NP, L3-4	31.9 ± 0.068	21.9 ± 0.112	38.1 ± 0.102	1.6 ± 0.094	0.5 ± 0.035	6.1 ± 0.017
6, female, NP, L3-4	45.6 ± 0.056	26.7 ± 0.106	26.1 ± 0.103	0.9 ± 0.088	0.4 ± 0.029	0.3 ± 0.052
7, female, NP, L4-5	47.9 ± 0.056	27.1 ± 0.107	23.4 ± 0.104	0.7 ± 0.087	0.5 ± 0.026	0.3 ± 0.047
16, male, NP, L4-5	47.2 ± 0.053	28.0 ± 0.109	23.7 ± 0.106	0.5 ± 0.093	0.3 ± 0.032	0.4 ± 0.047

**Fig. 6:**
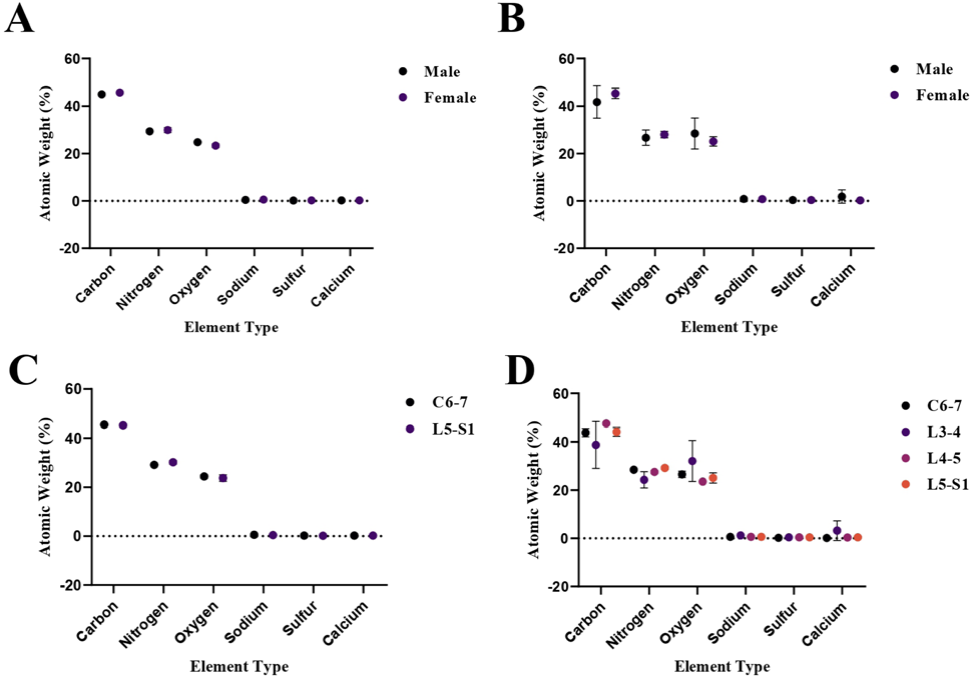
Gender and location based elemental composition percentages presented in Table 2. (A) Gender-based elemental compositions of AF tissues, (B) gender-based elemental compositions of NP tissues, (C) location-based elemental compositions of AF tissues, (D) location based elemental compositions of NP tissues.

Gender and location based elemental composition percentages presented in Table 2, for both the AF and NP tissues, are shown in Fig. 6.

### 2.3 Statistical analysis results

**Fig. 7:**
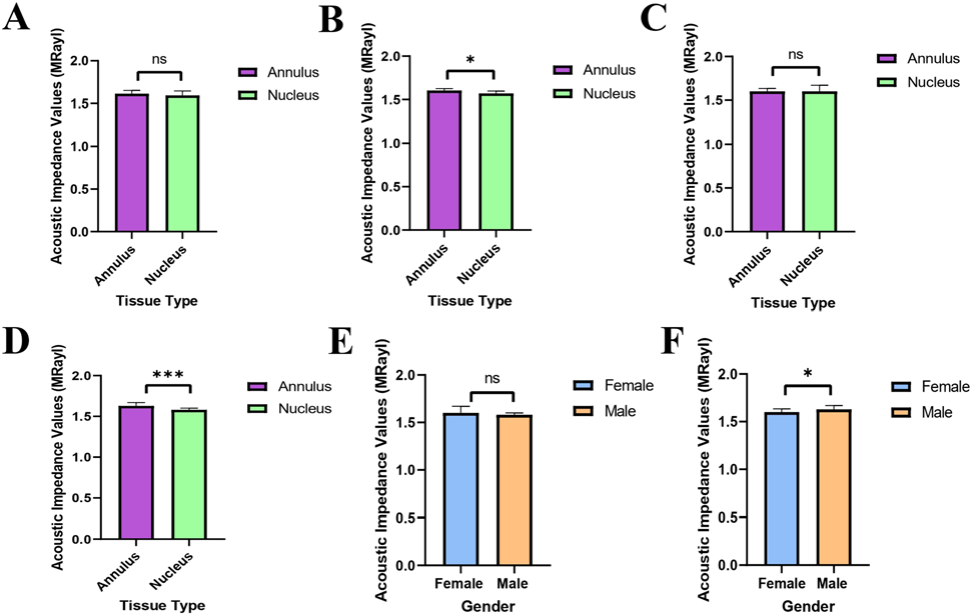
Statistical analyses of acoustic impedance values. (A) Analysis of all acoustic impedance values of both AF and NP tissue samples. (B) Analysis of acoustic impedance values of the tissue samples that were examined with SEM imaging and EDS. (C) Analysis of female AF and NP tissues' acoustic impedance values. (D) Analysis of male AF and NP tissues' acoustic impedance values. (E) Gender-based analysis of the NP tissue samples' acoustic impedance values. (F) Gender-based analysis of the AF tissue samples' acoustic impedance values. (ns: non-significant, *p < 0.05, **p < 0.01, ***p < 0.001).

The statistical analyses of the acoustic impedance values were done with the GraphPad (Prism8) program for all the AF and NP tissues and for tissues that were examined also with EDS and SEM imaging. The graphs generated are presented in Fig. 7.

## 3 Discussion

SAM is successful in monitoring the mechanical properties of AF and NP tissues, by calculating the acoustic impedance values (Table 1). As can be seen in Table 1 and Fig. 2, in most of the patients, acoustic impedance values of AF tissues are greater than those of NP tissues, due to the fact that AF is a fibrocartilaginous tissue composed of highly cross-linked collagen fibrils, whereas NP is more amorphous with a small percentage of randomly oriented fibrils.^26^Fig. 4 shows the difference in structures of AF and NP. However, in some of the patients NP tissues are stiffer than AF tissues. This can be a result of higher calcium level in NP tissues, as can be observed in patient 5 (Table 2 and Fig. 5B). This male patient has a stiffer NP tissue from L3-4 disc with a high acoustic impedance value (Table 1). Calcium deposits in the spine can be due to aging, infections or some treatments. Even though, calcifications are found to be the biomarker of disc degeneration, the mechanisms for calcium deposits are rarely studied.^27^ Besides, even though intervertebral disc calcification (IDC) is common in elderly people, in males, the frequency of IDC was found to be higher.^28^ In this study, we did not know the ages of the patients, therefore, in a further study, age and gender correlations with calcification can be studied since it is known that age has a significant impact on herniation in general and in IVDH.^29–31^ In this study, for EDS analysis the AF and NP tissues were dried with CO_2_ gas, which is used in critical drying and opens the pores on the tissue surface, allowing the water inside the tissue to escape.^32^ During this process, calcium (Ca) is transported to the surface.^32^ Ca that rises to the surface under high CO_2_ pressure undergoes carbonization.^32,33^ Since the binding energy of Ca is lower than the binding energy of nitrogen (N), oxygen tends to bond with Ca.^34^ This explains the increase in the amount of Ca and the decrease in the amount of N in the EDS analysis.^35^ Since all the tissues go under the same process before the EDS analysis, the effects of the CO_2_ would be similar on the tissues, therefore, we assumed that this kind of difference can be ignored. Acoustic impedance maps (Fig. 1) were generated from the intensity maps of samples in AI mode of SAM. These intensity images of stiffer surfaces were brighter than the ones of softer surfaces, due to the fact that the brightness of intensity images directly depends on the ultrasound intensity reflected, which is greater from a stiffer surface. Therefore, a different component, such as calcification, with different elasticity value can be distinguished within tissues with micrometer resolution by SAM. The calcium level in patient 5 was found to be the highest with EDS analysis, as can be seen in Table 2 and Fig. 5B. This increased level of calcification made this tissue stiffer and therefore, increased its acoustic impedance value. Oxygen and sodium levels were also higher for this patient when compared with others. On the other hand, carbon and nitrogen levels were lower than the ones of other patients' tissues. Disc degeneration can be due to nutrient transport disruption which can result from many phenomena such as lack of motion, high frequency loading, disk injury, aging or smoking.^36^ However, the mechanism behind nutrition delivery is very complex. Glucose is the main energy supply for the disc.^37^ Oxygen is vital for proper cell function.^38^ Mineralization was detected in degenerated discs, especially in the specimens that exhibited calcification,^39^ therefore, higher sodium level in patient 5 may be a result of this. For patient 5 with highly calcified NP tissue, we assume that all element levels differ from other patients' element levels due to greatly altered nutrition transport and can further be studied with more patients.

In Fig. 2, the female NP tissues' acoustic impedance values are not as homogenously distributed as the values of other tissues of male patients and female AF tissues, making the standard deviation (error bar) greater. This might result from their menopausal status, which changes estrogen level abruptly and some studies show that menopause increases IVDH.^40,41^ Also, it is shown that menopausal status is correlated with lumbar disc's mineral density.^42^ In this study, we did not have the age information of the patients and the menopausal status of the female patients, because of that, the correlation between the acoustic impedance values of the female AF and NP tissues and the calcification depending on the menopausal status could not be investigated but should be further examined.

In Fig. 3, a highly calcified NP tissue of patient 5 was compared with another patient's (Patient 1) NP tissue. It is also obvious in this figure that patient 5 has very high calcification level.

Table 1 shows herniated disc positions in this study as C3-4, C6-7, L3-4, L4-5 and L5-S1. Most of the patients have herniation in the lower lumbar spine, especially between the fourth and fifth lumbar vertebrae and between the fifth lumbar vertebra and the first sacral vertebrae (the L4-5 and L5-S1 levels), which is in agreement with literature.^43^ For a correlation between position and acoustic impedance value the number of patients, both male and female, has to be increased.

As can be seen in Fig. 7, the acoustic impedance values of all data were scientifically non-significant for AF and NP tissues (Fig. 7A), this can be a result of the fact that the gender distribution was not equal in the data. However, when the acoustic impedance values AF and NP tissues, that were examined with SEM and EDS, were analyzed, it was seen that the impedance values of AF tissues were significantly higher than the NP tissues (Fig. 7B). The acoustic impedance values of female tissues were non-significant (Fig. 7C), whereas the acoustic impedance values of male tissues were highly significant (Fig. 7D) indicating that the male AF tissues' impedance values were significantly higher than male NP tissues' impedance values. There was no scientifically significant difference between the female and male NP tissues' acoustic impedance values (Fig. 7E). On the other hand, the male AF tissues' acoustic impedance values were significantly higher than the female AF tissues' impedance values (Fig. 7F).

Since it is known that disorganization of collagen fibers leads to disc degeneration, therefore may lead to or accelerate the herniation of the intervertebral disc, the collagen protein organizations can also be examined with imaging approaches.^44^

## 4 Conclusions

In this study, we aimed to examine AF and NP tissues of female and male patients with cervical and lumbar intervertebral disc herniation, by SAM and SEM-EDS. By SAM, we managed to observe acoustic property variations within AF and NP tissues from female and male patients by obtaining acoustic impedance maps. We determined higher resolution images together with chemical information of the tissues by SEM-EDS analysis. Higher calcification in a tissue caused higher acoustic impedance value obtained by SAM and altered element levels obtained by SEM-EDS. Consequently, we can say that the AF and NP tissue variations in intervertebral disc herniation patients are observed by SAM and SEM-EDS for the first time and this achievement may result in combining these techniques in the future for the investigation and removal of the herniated AF and NP tissues with micrometer resolution.

## 5 Materials and methods

### 5.1 Ethics declaration

This study was ethically approved by Istanbul Biruni University Ethics Committee (Number: 2020/44-11) and informed consent was obtained from each participant. All experiments were performed in accordance with relevant guidelines and regulations.

### 5.2 Specimens

All patients were operated under general anesthesia. The standard surgical technique for discectomy was utilized for obtaining the specimens. Briefly, for the lumbar spine, the access was established through a midline incision, paravertebral muscle dissection from the posterior aspect of the spine and facetectomy at the index level. Once the exiting and traversing nerve roots were identified, the disc joint was exposed in the Kambin triangle. For the cervical spine, right sided transverse incision was performed and the intervertebral disc was approached initially by dissection though the medial side of the sternocleidomastoid muscle and later between the carotid artery and esophagus. Once the disc was exposed and verified, annulus fibrosus was cut with a scalpel and a rectangular specimen was obtained. The nucleus pulposus specimen was taken by using surgical curettes and pituitary rongeurs through the established window. Care was taken to preserve the mechanical integrity of the specimens. Electrocautery was not employed in order to avoid thermal damage to the tissues.

### 5.3 Scanning acoustic microscopy

**Fig. 8:**
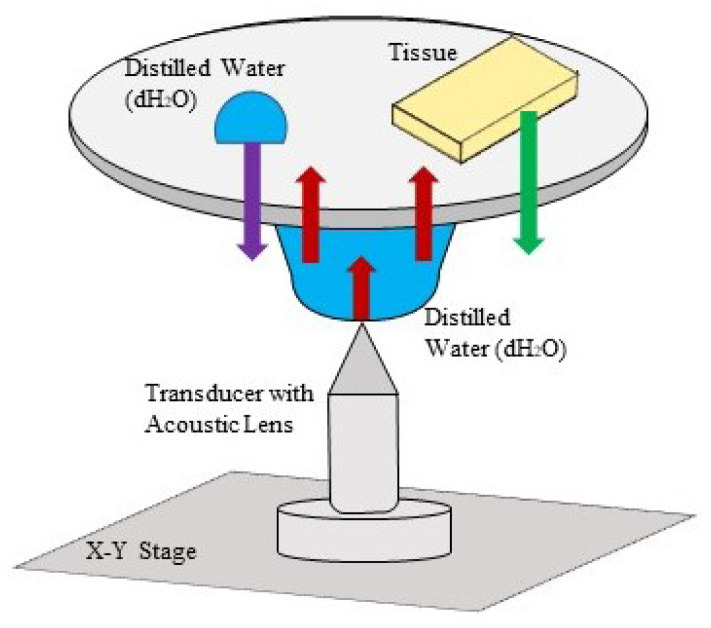
Working principle of scanning acoustic microscopy (SAM) in acoustic impedance (AI) mode for acquiring the acoustic impedance values of annulus nucleus (AF) and nucleus pulposus (NP) tissues. The red arrow presents the generated ultrasound signal by the transducer, the purple arrow presents the ultrasound signal reflected back from the reference, the green arrow presents the ultrasound signal reflected back from the tissue surface.

Scanning acoustic microscope (AMS-50SI) used in acoustic impedance experiments is developed by Honda Electronics (Toyohashi, Japan). Acoustic impedance (AI) mode chosen for the characterization is shown in Fig. 8. 80 MHz transducer was the ultrasonic signal generator and receiver in this study. 80 MHz transducer has a focal length of 1.5 mm with a spot size of 17 μm. The coupling medium between the quartz lens and the substrate is distilled water. The generated ultrasonic signals are scanned by the *X*–*Y* stage and the reflected signals both from the reference and the target material are compared and analyzed to generate the intensity and acoustic impedance maps of the region of interest with 300 × 300 sampling points with a lateral resolution of approximately 20 μm.^45^

### 5.4 Scanning electron microscopy and energy dispersive spectroscopy

AF and NP tissue samples excised from the patients were kneaded in sizes not exceeding 0.5 cm and the kneaded tissue pieces were exchanged 10 times and were dried under carbon dioxide using acetone in a critical point dryer, Leica© EM CPD300 Critical Point Dryer device, to remove the water contained in them, and the critical drying was completed at 40 °C without damaging the tissues. This process was done to prevent damage to tissues because of sudden evaporation of water when exposed to high vacuum. The samples were placed on carbon tapes adhered to the aluminum mount for SEM imaging and EDS analysis. Approximately 70 nm carbon coating was applied with 3 flash pulses in the Leica EM ACE 200 device before the tissues were taken from the critical dryer and placed in SEM. With this process, a conductive layer is created on the surface of the tissues, preventing the formation of an electron cloud. By this layer, the number of electrons coming to the secondary electron detector and the number of X-rays coming to the EDS detector increase and therefore, clearer SEM images are obtained and more reliable and accurate measurements are done in EDS analysis. Another process for increasing the number of signals per second in EDS analysis is to increase the voltage applied to the filament, that increases the speed of the electrons. These primarily accelerated electrons carry the electrons in the 2nd and 3rd orbits of the atoms within tissues to the upper energy level orbits, and the X-rays emitted by them have characteristic features. To obtain 5000 or more counts per second from an insulating surface, the spot aperture of the beam was increased by applying 20 kV (spot size 5.5 for Thermo Scientific™ Quattro ESEM). The visualization of the tissues was done under a high vacuum.

Elemental analyses were made with the EDAX brand Energy Dispersive Spectroscopy (EDS) detector. Before analyses, the EDAX EDS device was calibrated with Al and Cu standard samples with APEX software automatically, and EDS analysis was performed for each tissue sample from a suitable area at 1000 times magnification, 10 mm working distance for 14 minutes. The spectra of carbon (C), nitrogen (N), oxygen (O), sodium (Na), sulfur (S), and calcium (Ca) were measured in percentage by weight. The purpose of performing area analysis instead of point or line analysis was to calculate the average elemental weights of different structures within the tissue. By this way, the results obtained from a greater area were more significant and accurate.

### 5.5 Statistical analysis

Using the GraphPad (Prism8) program, statistical analysis of acoustic impedance values of AF and NP tissues of both genders were performed. The graphics of the analyzed data were generated from the GraphPad (Prism8) program. Unpaired-one-tailed Student's *t*-test (*t*-test with unequal variance) was used for the determination of statistically significant differences in acoustic impedance measurements among different genders, tissue types, calcific-rich and less calcific areas in samples and the level of statistical significance level was set to *p* < 0.05.

## Author contributions

B. T., B. D. and S. B. conducted the experiments, B. T. and B. D. analysed the results, K. A. and C. O. performed the excisions, M. B. U. acquired funding and supervised. All authors reviewed the manuscript.

## Conflicts of interest

There are no conflicts to declare.

## Supplementary Material
